# Tailoring a Heterogeneous Bimodal Structure for Superior Strength–Ductility Synergy in Dilute Mg-0.4Al-0.3Ca-0.2Mn-xSn Alloy: The Critical Role of Trace Sn Microalloying

**DOI:** 10.3390/ma19030507

**Published:** 2026-01-27

**Authors:** Guo Li, Jiahao Zhang, Li Sun, Xinyang Ge, Bin Li, Guobing Wei

**Affiliations:** 1 College of Materials Science and Engineering, Chongqing University, Chongqing 400044, Chinagexinyangcqu@163.com (X.G.); 2 School of Industrial Engineering, Ningxia Vocational and Technical University, Yinchuan 750021, China; 3 National Key Laboratory of Advanced Casting Technologies, Chongqing University, Chongqing 400044, China; 4 International Joint Laboratory for Light Alloys, Ministry of Education, Chongqing University, Chongqing 400044, China; 5 Intelligent Manufacturing and Automobile School (Aeronautics School), Chongqing Polytechnic University of Electronic Technology, Chongqing 401331, China

**Keywords:** Mg alloys, trace Sn, hot extrusion, heterogeneous bimodal structure, particle-stimulated nucleation, strength-ductility synergy

## Abstract

To achieve an optimal balance of mechanical properties in low-cost alloy systems, this study tailored a heterogeneous bimodal structure in dilute Mg-0.4Al-0.3Ca-0.2Mn-xSn alloys (x = 0, 0.1 wt.%) and systematically investigated the critical role of trace Sn microalloying during hot extrusion. Mg-0.4Al-0.3Ca-0.2Mn-xSn alloys were fabricated via melting, homogenization, and subsequent hot extrusion at 320 °C. Trace Sn addition induced the formation of uniformly distributed CaMgSn phases within the homogenized matrix, facilitating a synergistic enhancement of strength and ductility. Specifically, the extruded alloys exhibited a characteristic bimodal grain structure consisting of coarse un-dynamic recrystallized (unDRXed) grains and fine dynamic recrystallized (DRXed) grains. Sn microalloying effectively refined the DRXed grains from 2.66 μm to 2.11 μm and significantly boosted the elongation (EL) from 12.9% to 26.3% while maintaining an Ultimate Tensile Strength (UTS) of 274 MPa. The Sn-containing secondary phases served as potent sites for particle-stimulated nucleation (PSN), thereby promoting the DRX process and reducing the texture intensity from 20.89 to 9.99. Overall, the superior strength-ductility synergy is primarily governed by the formation of the heterogeneous bimodal structure, where trace Sn facilitates grain refinement and texture weakening through PSN mechanisms, providing a robust strategy for the design of high-performance dilute magnesium alloys.

## 1. Introduction

The global materials engineering landscape is currently undergoing a profound transformation driven by the urgent necessity of “carbon neutrality” and stringent environmental regulations [1]. As the automotive, aerospace, and rail transportation sectors grapple with the dual mandate of reducing greenhouse gas emissions and enhancing fuel efficiency, the demand for lightweight structural materials has intensified unprecedentedly [2,3]. In this context, Magnesium (Mg) alloys have emerged as a strategic material of choice. Distinguished as the lightest structural metal—approximately 33% lighter than aluminum and 77% lighter than steel—Mg offers immense potential for realizing significant weight reduction in structural components [4]. Beyond their low density, Mg alloys possess high specific strength, exceptional damping capacity, electromagnetic shielding properties, and excellent recyclability, positioning them as a cornerstone for the next generation of eco-friendly transport solutions [5,6].

However, the widespread industrial adoption of Mg alloys is currently impeded by significant metallurgical bottlenecks. Foremost among these is the inherent trade-off between strength and ductility, a challenge rooted in the hexagonal close-packed (HCP) crystal structure of Mg [7]. At ambient temperatures, the deformation of Mg is dominated by basal <a> slip and {10-12} extension twinning, which provide insufficient independent slip systems to satisfy the von Mises criterion for homogeneous deformation [8]. Consequently, thermomechanically processed Mg alloys, particularly those subjected to rolling or extrusion, typically develop strong basal textures where the crystallographic c-axes align perpendicular to the deformation direction [9]. This strong texture results in significant mechanical anisotropy and poor room-temperature formability. While alloying with Rare Earth (RE) elements (e.g., Gd, Y, Nd) has proven effective in weakening these textures and boosting strength through solute clustering and precipitation hardening, the prohibitive cost and strategic scarcity of RE elements render them economically unviable for high-volume, cost-sensitive applications like mass-market automotive components [10,11]. Therefore, the development of high-performance, low-cost, RE-free “dilute” Mg alloys has become a critical objective for the materials science community.

To overcome the strength–ductility paradox without resorting to expensive alloying elements, the concept of “heterogeneous structure design” has emerged as a robust and innovative strategy [12]. Unlike traditional ultrafine-grained materials which often sacrifice ductility for strength due to limited work-hardening capacity, heterogeneous structures—specifically bimodal grain size distributions—can achieve a synergistic enhancement of both properties [13,14]. A heterogeneous bimodal structure typically comprises an alternating arrangement of coarse, unDRXed grains and fine, DRXed grains [15]. This architecture leverages the high yield strength of the fine “hard” domains while capitalizing on the superior dislocation storage capacity and plasticity of the coarse “soft” domains [16]. The mechanical incompatibility between these domains during deformation generates Geometrically Necessary Dislocations (GNDs) at the hetero-interfaces, resulting in significant Hetero-Deformation Induced (HDI) strengthening (back stress) which sustains work hardening and delays necking [17]. Achieving such a tailored microstructure, however, requires a delicate balance of alloy composition and thermomechanical processing parameters, particularly during hot extrusion.

Microalloying serves as an effective and economical approach to tailor the microstructures of Mg alloys for such heterogeneous designs. In the context of RE-free systems, the Mg-Al-Ca-Mn (AXM) family has shown great promise. Trace Aluminum is known to reduce the anisotropy of the Critical Resolved Shear Stress (CRSS) between basal and non-basal slip systems, thereby enhancing intrinsic ductility [18,19]. The synergistic addition of Calcium (Ca) facilitates the formation of thermally stable phases (such as Al_2_Ca or (Mg, Al)_2_Ca), which effectively pin grain boundaries via the Zener drag mechanism, preserving the necessary unDRXed skeletal structure during hot working [19,20]. Furthermore, trace Manganese (Mn) additions contribute to grain refinement and corrosion resistance through the formation of Mn-containing precipitates or G.P. zones, which can also influence dynamic recrystallization kinetics [21,22].

Recently, Tin (Sn) has gained significant attention as a promising microalloying element for Mg. Theoretical and experimental studies suggest that Sn can reduce the Stacking Fault Energy (SFE) of the Mg matrix, a critical factor in promoting the accumulation of dislocations and activating non-basal slip modes such as prismatic or pyramidal <c+a> slip [23,24]. Lower SFE suppresses cross-slip and dynamic recovery, encouraging work hardening. Moreover, in Ca-containing systems, Sn exhibits a strong affinity for Ca, leading to the formation of specific intermetallic phases like CaMgSn. These phases are distinct from Al-Ca precipitates; if engineered correctly in terms of size and distribution, CaMgSn particles can serve as potent sites for PSN during dynamic recrystallization [25]. PSN is a key mechanism for texture weakening, as grains nucleated at particles often possess randomized orientations, thereby diluting the strong basal texture inherent to extrusion.

Despite the individual benefits of Al, Ca, Mn, and Sn being documented, the specific role of trace Sn microalloying in tailoring a heterogeneous bimodal structure within the dilute Mg-Al-Ca-Mn system remains underexplored. Specifically, the interplay between Sn-induced phase transformation (formation of CaMgSn), the PSN mechanism, and the resulting evolution of HDI strengthening has not been fully elucidated for this specific alloy class.

In this study, a dilute Mg-0.4Al-0.3Ca-0.2Mn-xSn alloy (x = 0, 0.1 wt.%) was designed and fabricated via hot extrusion at a relatively low temperature of 320 °C. We systematically investigated the influence of trace Sn on the evolution of second-phase particles, DRX behavior, and texture modification. Specifically, the role of Sn-containing phases in stimulating recrystallization via the PSN mechanism and the resulting heterogeneous bimodal structure were analyzed. This work aims to provide a fundamental understanding of how trace Sn microalloying facilitates an exceptional strength-ductility balance, offering a new pathway for the design of high-performance RE-free Mg alloys.

## 2. Materials and Methods

### 2.1. Alloy Preparation and Casting

The Mg-0.4Al-0.3Ca-0.2Mn-xSn (x = 0, 0.1 wt.%) alloys were fabricated via a conventional melting and gravity casting route. The raw materials included high-purity Mg ingots (≥99.9%), Al ingots (≥99.9%), Sn particles (≥99.9%), and master alloys of Mg-20Ca (wt.%) and Mg-5Mn (wt.%). Melting was conducted in an SJ 2-5 electrical resistance furnace with a rated power of 5 kW. To prevent oxidation and ignition of the magnesium melt, a protective atmosphere of SF_6_ and CO_2_ (volume ratio of approximately 1:100) was continuously maintained throughout the process.

Before melting, the stainless steel crucibles (ø 90 mm × 280 mm) and raw materials were preheated at 300 °C to ensure the complete removal of surface moisture. High-purity Mg was first melted at 720 °C and held for 10 min. Subsequently, pre-calculated amounts of Al, Mg-5Mn, and Mg-20Ca master alloys were introduced and held for 20–30 min to achieve chemical homogeneity. Finally, trace Sn particles were added and held for an additional 10 min. Following mechanical stirring and slag removal using boron nitride-coated tools, the melt was subjected to water-cooled quenching to induce rapid solidification and obtain a fine-grained as-cast microstructure. This process yielded cylindrical ingots with dimensions of ø 80 mm × 190 mm.

### 2.2. Homogenization and Hot Extrusion

The as-cast ingots were machined to remove surface oxide layers. Following this, a homogenization heat treatment was conducted at 350 °C for 12 h; upon completion, the ingots were immediately extracted and subjected to water quenching to cool them rapidly to room temperature. Subsequently, the samples were hot-extruded at 320 °C with an extrusion ratio of approximately 25:1 (reducing the diameter from ø 80 mm to ø 16 mm) and a constant ram speed of 25 mm/s.

### 2.3. Microstructural and Mechanical Characterization

The microstructures of the homogenized and as-extruded alloys were characterized using optical microscopy (OM, OLYMPUS PMG3, OLYMPUS, Tokyo, Japan) and scanning electron microscopy (SEM, JEOL JSM-7800F, JEOL Ltd., Tokyo, Japan). To ensure that the observations represented the inherent microstructural characteristics of the bulk materials, all characterization specimens were extracted from the central regions of the homogenized ingots and extruded bars via wire electrical discharge machining.

Specimens were ground with SiC papers (up to 3000 grit) and polished with diamond pastes. For OM and SEM characterization, the polished surfaces were etched using a picric-acetic acid solution. For EBSD analysis (JEOL JSM-7800F, JEOL Ltd., Tokyo, Japan), the samples underwent an additional electropolishing step after mechanical polishing to eliminate surface residual stress and ensure high indexing rates. Crystallographic features, including grain orientation, local misorientation, Schmid factor (SF) distribution, and texture evolution, were systematically examined via EBSD, while elemental distributions were analyzed using energy-dispersive X-ray spectroscopy (EDS). The average grain size was statistically calculated from EBSD data using the equivalent circle diameter method, with high-angle grain boundaries defined by a misorientation angle >15°.

Finally, mechanical performance was evaluated by testing sub-size tensile specimens (gauge width: 8 mm; thickness: 2 mm) extracted along the extrusion direction. Although sub-size specimens were utilized due to sample dimensions, the testing procedures and data acquisition strictly adhered to the principles of the ASTM E8/E8M standard [26]. Tensile tests were conducted using an electronic universal testing machine (CMT-5105, SANS Testing Machine Co., Ltd., Shenzhen, China) at a room-temperature initial strain rate of 10^−3^ s^−1^.

## 3. Results and Discussion

### 3.1. Impact of Trace Sn on CaMgSn Precipitation

**Figure 1:**
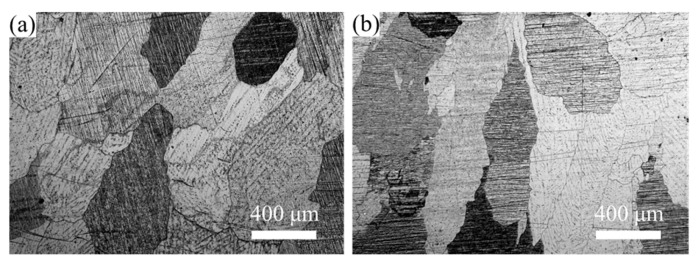
OM of homogenized Mg-0.4Al-0.3Ca-0.2Mn-xSn alloys (x = 0, 0.1 wt.%): (**a**) x = 0; (**b**) x = 0.1.

Figure 1 displays the OM of the Mg-0.4Al-0.3Ca-0.2Mn-xSn alloys (x = 0, 0.1 wt.%) after homogenization at 350 °C for 12 h. It can be observed that after the prolonged heat treatment, both alloys exhibit a characteristic microstructure composed of relatively coarse equiaxed grains. No significant difference in the grain morphology was observed between the base alloy and the Sn-containing alloy in the homogenized state.

**Figure 2:**
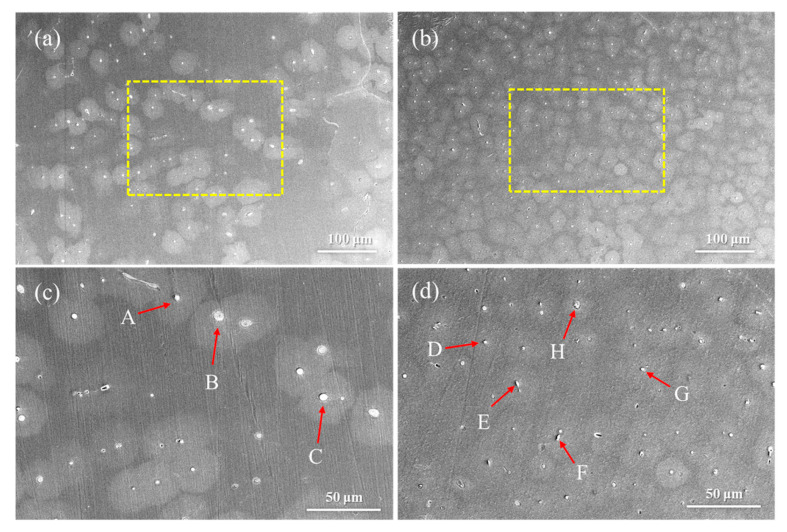
SEM images of homogenized Mg-0.4Al-0.3Ca-0.2Mn-xSn alloys (x = 0, 0.1 wt.%) at different magnifications: (**a**,**c**) x = 0; (**b**,**d**) x = 0.1.

Figure 2 shows the SEM images of homogenized Mg-0.4Al-0.3Ca-0.2Mn-xSn alloys (x = 0, 0.1 wt.%) at different magnifications, while Table 1 presents the EDS results of the second-phase particles marked by arrows in Figure 2. Phase identification is based on thermodynamic principles established for Mg-rich systems. In the Mg-Ca-Sn system, the Sn/Ca ratio determines the solidification sequence; specifically, when this ratio is less than 3, the formation of the Mg_2_Sn phase is suppressed in favor of the CaMgSn ternary phase [27]. This condition aligns with our trace Sn addition, for which the Sn/Ca ratio remains well below the threshold. Furthermore, based on the research of Suzuki [28], (Mg, Al)_2_Ca is the predominant secondary phase when the Ca/Al ratio ranges between 0.7 and 1.64. Given that the Ca/Al ratio in our alloy is approximately 0.75, particles B, C and H are identified as the (Mg, Al)_2_Ca, which exhibits a rounded morphology. Moreover, particles A exhibit characteristic bright square morphology and Mn-enriched composition, which is typical of the Al_8_Mn_5_ phase often observed in Mg-Al-Mn alloys [29]. The formation of these intermetallic compounds is driven by the electronegativity differences between the constituent elements [30]. With electronegativity values of 1.31, 1.61, 1.00 and 1.96 for Mg, Al, Ca, and Sn, respectively, the substantial electronegativity gap between Sn and Ca facilitates the formation of the high-melting-point CaMgSn phase. EDS analysis confirms that the irregular white particles (D, G) and bright short-rod particles (E, F) correspond to the CaMgSn phase. The precipitation temperature of (Mg, Al)_2_Ca phase is about 528 °C, and the precipitation temperature of CaMgSn phase is about 630 °C [31]. In addition, due to the similarity in size between Al and Mg atoms, Al elements are easily enriched near the CaMgSn phases. The CaMgSn phases form before the (Mg, Al)_2_Ca phases and serve as a heterogeneous nucleation point to promote the nucleation of the (Mg, Al)_2_Ca phase, resulting in a higher Al content in the CaMgSn phase (Table 1). The homogenization temperature in this study is 350 °C; thus, the coarse CaMgSn phase decomposes into smaller second-phase particles that are dispersed in the matrix after heat treatment. No Mn-containing secondary phase was observed in the alloy, because the Mn-containing secondary phase is relatively small or Mn is solubilized in the Mg alloy matrix [22].

### 3.2. Tailoring Heterogeneous Bimodal Structure via Particle-Stimulated Nucleation

**Figure 3:**
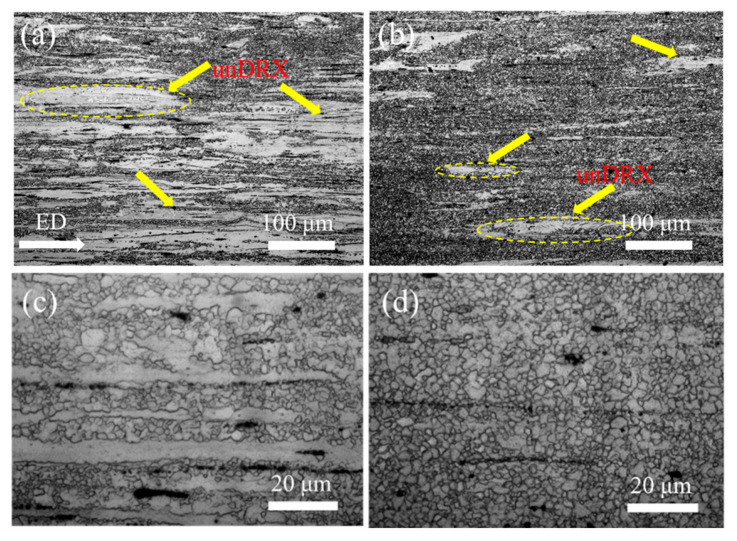
Microstructures of Mg-0.4Al-0.3Ca-0.2Mn-xSn alloys (x = 0, 0.1 wt.%) after extrusion at different magnifications: (**a**,**c**) x = 0; (**b**,**d**) x = 0.1.

Figure 3 shows OM micrographs of homogenized and extruded Mg-0.4Al-0.3Ca-0.2Mn-xSn alloys (x = 0, 0.1 wt.%). Following extrusion, the grain structure undergoes pronounced refinement, transitioning from coarse equiaxed grains to a heterogeneous bimodal distribution consisting of fine DRXed grains and elongated unDRXed grains [24]. Statistical analysis reveals that the unDRXed volume fraction decreases sharply from 60% in the Sn-free alloy to 14% in the 0.1 wt.% Sn-alloy. This enhanced DRX degree is visually evident in Figure 3b,d, where DRXed grains are observed clustering around fragmented secondary phases. The finely dispersed Sn-containing phases in the homogenized precursor act as potent sites for PSN during hot deformation.

**Figure 4:**
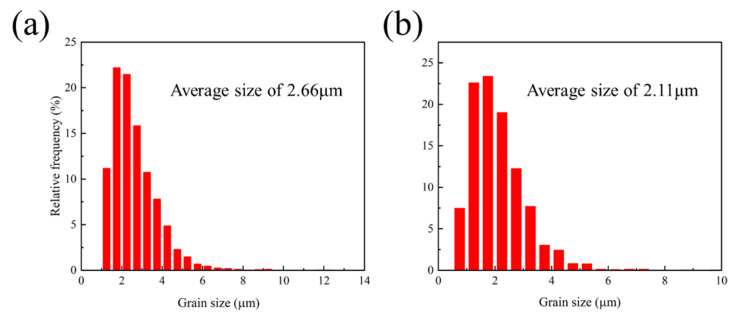
Grain size distribution of extruded Mg-Al-Ca-Mn-xSn alloys (x = 0, 0.1 wt.%): (**a**) x = 0; (**b**) x = 0.1.

Furthermore, Sn addition is known to reduce the SFE of Mg alloys, which facilitates the activation of non-basal slip systems and promotes dislocation accumulation. This increased dislocation density provides a higher driving force for DRX. As depicted in Figure 4, the average DRXed grain size is refined from 2.66 μm (x = 0) to 2.11 μm (x = 0.1).

**Figure 5:**
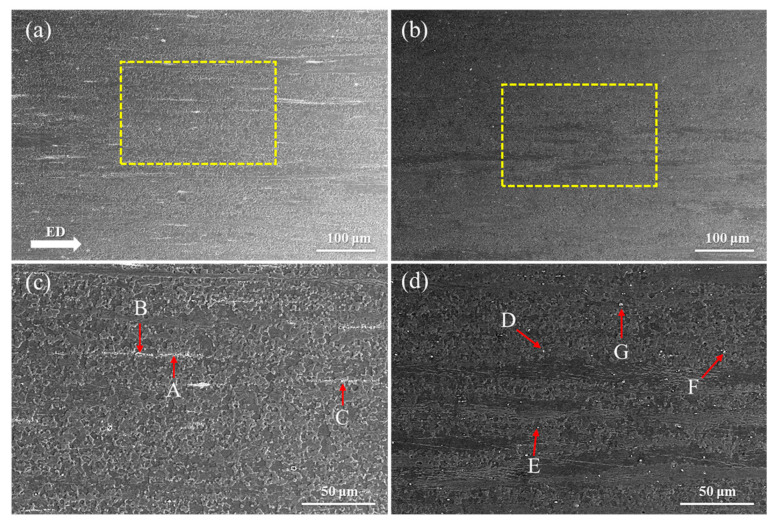
SEM images of extruded Mg-0.4Al-0.3Ca-0.2Mn-xSn alloys (x = 0, 0.1 wt.%) at different magnifications: (**a**,**c**) x = 0; (**b**,**d**) x = 0.1.

As illustrated in Figure 5, the second-phase particles undergo significant fragmentation during extrusion, becoming refined and dispersed along the extrusion direction (ED) (Figure 5a–d). SEM and EDS characterizations (Figure 5 and Table 2) confirm that the intermetallic compounds—(Mg, Al)_2_Ca (particles A, B, G), Al_8_Mn_5_ (particle C) and CaMgSn (particles D, E, F) —correspond to the phases observed in the homogenized alloy. These phases are crushed into smaller fragments and aligned into stringers along the ED. Specifically, the (Mg, Al)_2_Ca phase is broken into dense, fine particles with narrow inter-particle spacing, forming linear arrays at grain boundaries. Notably, the 0.1 wt.% Sn-containing alloy exhibits a more uniform distribution of these secondary phases compared to the Sn-free counterpart (Figure 5). These fragmented particles not only increase the local stored deformation energy but also serve as potent sites for PSN. Consequently, the presence of these particles accelerates the DRX kinetics and increases the DRX fraction, ultimately contributing to the observed grain refinement.

### 3.3. Texture Weakening and Grain Orientation Randomization

**Figure 6:**
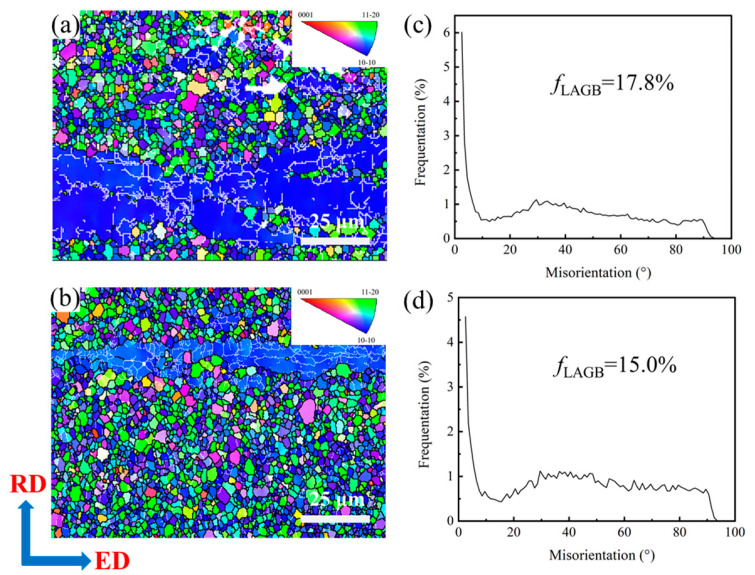
IPF maps and misorientation angle distributions of extruded Mg-0.4Al-0.3Ca-0.2Mn-xSn alloys (x = 0, 0.1 wt.%): (**a**,**c**) x = 0; (**b**,**d**) x = 0.1.

Figure 6a,b illustrate the Inverse Pole Figure (IPF) maps of the extruded Mg-0.4Al-0.3Ca-0.2Mn-xSn alloys (x = 0, 0.1 wt.%). Both alloys exhibit a characteristic bimodal microstructure, which is composed of fine DRXed grains and coarse, elongated un-DRXed grains. In the Sn-free alloy (Figure 6a), large areas of un-DRXed regions are observed, characterized by a predominant blue color. This indicates that their <10-10> directions are oriented parallel to ED, a common feature representing a strong basal fiber texture in extruded Mg alloys. With the addition of 0.1 wt.% Sn (Figure 6b), two significant changes are observed. First, the area fraction of DRXed grains increases markedly, and the continuous coarse-grain bands are effectively broken down or narrowed. Second, the Sn addition leads to a noticeable grain refinement within the DRXed regions compared to the base alloy. The random distribution of colors in the DRXed regions of both alloys suggests a relatively weak or randomized orientation compared to the un-DRXed regions. These results indicate that the minor addition of Sn promotes the dynamic recrystallization process and refines the microstructure, likely due to the solute drag effect or the formation of Sn-containing precipitates that pin the grain boundaries during extrusion.

Figure 6c,d illustrate the misorientation angle distributions of the extruded Mg-0.3Al-0.4Ca-0.2Mn-xSn (x = 0, 0.1) alloys. The fractions of low-angle grain boundaries (LAGBs) within the selected regions (corresponding to 2° ≤ θ ≤ 15°) are 17.8% and 15% for the alloys with x = 0 and x = 0.1, respectively. These results suggest that both alloys contain a high fraction of LAGBs, with the former exhibiting a marginally higher proportion primarily attributed to its greater content of unDRXed grains. Consistent with the IPF maps, a high density of LAGBs is concentrated within the unDRXed grains. Generally, a higher fraction of LAGBs correlates with an elevated dislocation density, which in turn enhances the strength and hardness of the alloy while reducing its plasticity and toughness.

**Figure 7:**
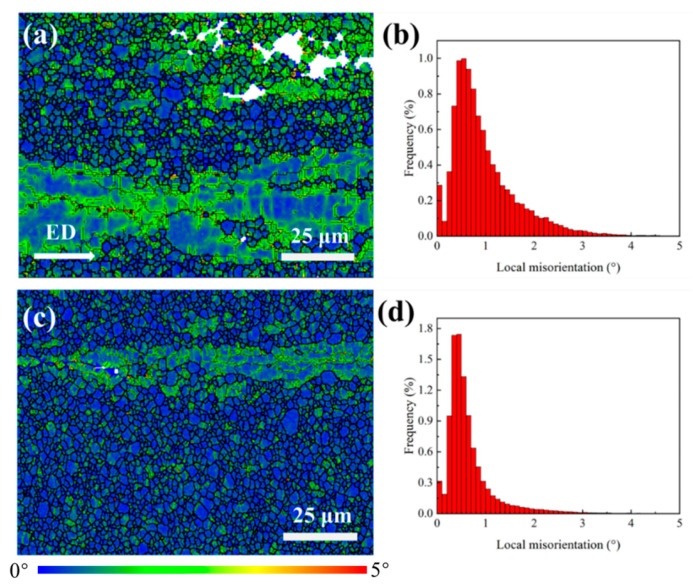
KAM maps and corresponding local misorientation angle distributions of extruded Mg-0.4Al-0.3Ca-0.2Mn-xSn alloys (x = 0, 0.1 wt.%): (**a**,**b**) x = 0; (**c**,**d**) x = 0.1.

The Kernel Average Misorientation (KAM) maps and the corresponding local misorientation angle distribution for the extruded Mg-0.4Al-0.3Ca-0.2Mn-xSn alloys are displayed in Figure 7. Generally, KAM values are positively correlated with the density of GNDs and the level of local lattice distortion. For the Sn-free alloy (Figure 7a), high KAM values (manifested as green-colored regions) are prominently concentrated within the interiors of the unDRXed elongated grains, indicating a substantial level of residual strain and dislocation accumulation. In contrast, the DRXed regions exhibit much lower KAM values (blue color), suggesting that the stored energy was significantly released during the DRX process.

Upon the addition of 0.1 wt.% Sn (Figure 7c), the overall KAM intensity decreases substantially. The area fraction of regions with high local misorientation is visibly reduced, which is consistent with the higher DRX fraction observed in this alloy. The statistical results in Figure 7b,d further support this observation. The peak frequency of the 0.1Sn alloy shifts toward a lower misorientation angle (~0.4°) compared to the base alloy, and the maximum frequency increases from approximately 1.0% to 1.7%. These results demonstrate that the Sn addition effectively promotes dynamic recrystallization, which in turn leads to a more efficient consumption of dislocations and a more uniform distribution of internal strain in the extruded alloy.

**Figure 8:**
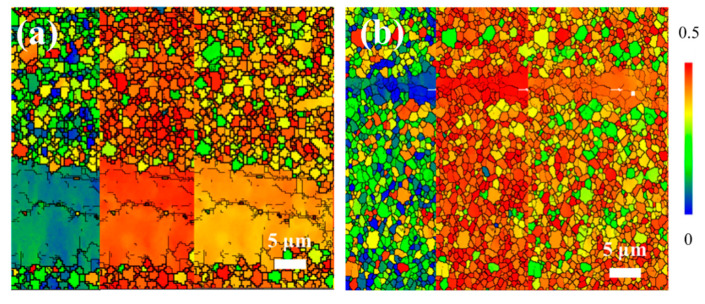
SF distributions maps of extruded Mg-0.4Al-0.3Ca-0.2Mn-xSn alloys (x = 0, 0.1 wt.%): (**a**) x = 0; (**b**) x = 0.1.

To further evaluate the effect of Sn addition on the plastic deformation behavior, the SF distribution maps for the basal {0001}<11-20>, prismatic {10-10}<11-20>, and pyramidal <c+a> slip systems of the extruded Mg-0.4Al-0.3Ca-0.2Mn-xSn alloys are illustrated in Figure 8. For the Sn-free alloy (Figure 8a), a distinct heterogeneity in SF values is observed between the DRXed and un-DRXed regions. Specifically, the coarse un-DRXed grains exhibit relatively low SF values for basal slip (indicated by the blue/green regions), suggesting a “hard orientation” that restricts basal slip activity during tensile or compressive deformation along the ED.

In contrast, the addition of 0.1 wt. % Sn (Figure 8b) leads to a more uniform and elevated SF distribution across the sampled area. This shift is primarily attributed to the significantly increased fraction of DRXed grains, which possess more randomized crystallographic orientations compared to the strong basal fiber texture of the un-DRXed regions. The higher average SF values for both basal and non-basal slip systems in the 0.1 Sn alloy imply that the CRSS can be surpassed at lower applied stresses, thereby facilitating slip initiation and enhancing the ductility of the alloy. These results suggest that Sn addition effectively “weakens” the sharp extrusion texture by promoting dynamic recrystallization, which contributes to a better balance between strength and ductility.

**Figure 9:**
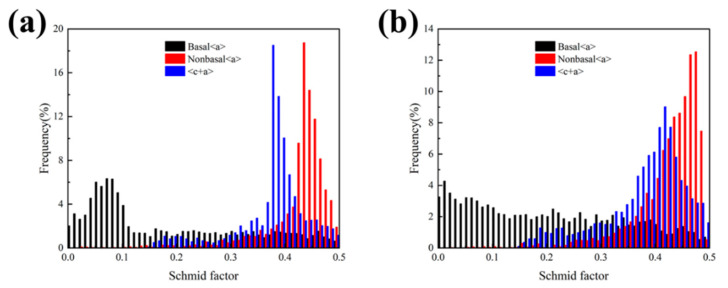
Frequency histograms of SF for basal <a>, non-basal <a>, and <c+a> slip systems in extruded Mg-0.4Al-0.3Ca-0.2Mn-xSn alloys (x = 0, 0.1 wt.%): (**a**) x = 0; (**b**) x = 0.1.

To quantitatively evaluate the ease of slip activation, Figure 9 presents the frequency histograms of SF for the basal <a>, non-basal <a>, and <c+a> slip systems. For the Sn-free alloy (Figure 9a), the SF for basal slip exhibits a pronounced bimodal distribution with a significant peak at low values (<0.1). This confirms that a large fraction of grains, particularly the coarse un-DRXed grains identified in Figure 8a, are in a “hard orientation” where the basal planes are nearly parallel to the ED. Such a configuration significantly restricts the activity of the primary basal slip system, leading to high yield strength but limited ductility. In contrast, the addition of 0.1 wt.% Sn (Figure 9b) results in a notable shift in the SF distribution toward higher values for all slip systems. The peak for basal slip at low SF values is substantially suppressed and redistributed toward the range of 0.2–0.5. Simultaneously, the frequency of high SF values (>0.4) for non-basal <a> and <c+a> slip systems increases markedly. This shift is directly correlated with the texture weakening induced by the higher degree of dynamic recrystallization (DRX) seen in Figure 8b. The increased average SF for both basal and non-basal slips implies that a lower external stress is required to reach the CRSS for multiple slip systems. Consequently, the 0.1Sn alloy promotes more homogeneous plastic deformation and enhanced ductility by facilitating the simultaneous activation of various slip modes.

**Figure 10:**
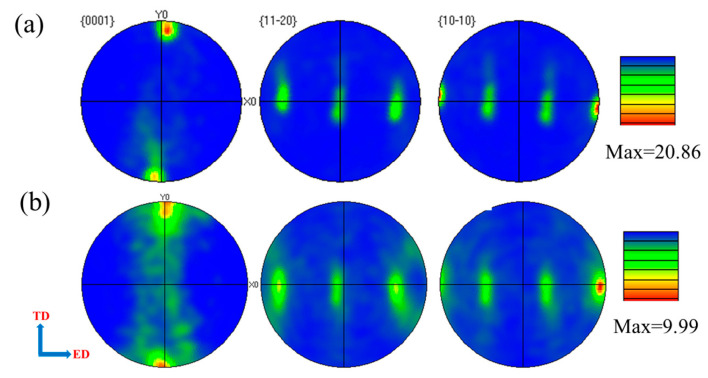
Pole figures of extruded Mg-0.4Al-0.3Ca-0.2Mn-xSn alloys (x = 0, 0.1 wt.%): (**a**) x = 0; (**b**) x = 0.1.

The macro-texture evolution of the extruded Mg-0.4Al-0.3Ca-0.2Mn-xSn alloys was further characterized using pole figures, as illustrated in Figure 10. Both alloys exhibit a typical basal fiber texture, where the {0001} basal poles are primarily distributed perpendicular to the ED. For the Sn-free alloy (Figure 10a), a high maximum texture intensity of 20.86 is observed, indicating a strong alignment of the basal planes parallel to the ED in the coarse un-DRXed regions. This strong texture coincides with the low SF values and high KAM previously discussed, suggesting significant plastic anisotropy and dislocation accumulation.

With the minor addition of 0.1 wt. % Sn (Figure 10b), the maximum texture intensity drastically decreases to 9.99, representing a significant texture-weakening effect. While the overall distribution pattern remains similar, the basal poles in Figure 10b show a higher degree of dispersion. This weakening is primarily attributed to the enhanced DRX process confirmed in Figure 6 and Figure 7. The newly formed DRXed grains possess more randomized orientations, which effectively dilute the sharp deformation texture of the original un-DRXed matrix. This reduced texture intensity directly accounts for the increased SFs for both basal and non-basal slips (Figure 9), thereby facilitating more balanced deformation modes and contributing to the superior ductility of the 0.1Sn alloy.

As evidenced by the IPF maps (Figure 6), the addition of 0.1 wt.% Sn effectively promotes dynamic recrystallization (DRX), leading to a higher area fraction of fine equiaxed grains that interrupt the continuous coarse un-DRXed bands. This heterogeneous configuration provides a unique combination of strengthening mechanisms: the fine DRXed grains contribute to strength via grain boundary strengthening, while the coarse un-DRXed regions, although exhibiting high dislocation density as shown in the KAM maps (Figure 7), serve as a skeleton to maintain high flow stress.

Meanwhile, the macro-texture analysis (Figure 10) reveals that trace Sn addition drastically reduces the maximum texture intensity from 20.86 to 9.99. This texture weakening is a direct consequence of the randomized orientations of the newly formed DRXed grains. The quantitative SF analysis (Figure 9) further clarifies the mechanical implications of this evolution. In the 0.1Sn alloy, the SF distributions for basal <a>, non-basal <a>, and <c+a> slips all shift toward higher values. Specifically, the increased frequency of high SF values for <c+a> pyramidal slip is critical, as it facilitates the accommodation of c-axis strain, which is often a bottleneck for the ductility of Mg alloys.

Therefore, trace Sn microalloying plays a dual role: it refines the microstructure by stimulating DRX and “softens” the hard-oriented grains by weakening the basal texture. This dual effect results in a more homogeneous distribution of internal strain and a lower threshold for activating multiple slip systems, thereby providing the possibility of achieving excellent plasticity while maintaining high strength.

### 3.4. Superior Strength–Ductility Synergy and HDI Strengthening Mechanisms

**Figure 11:**
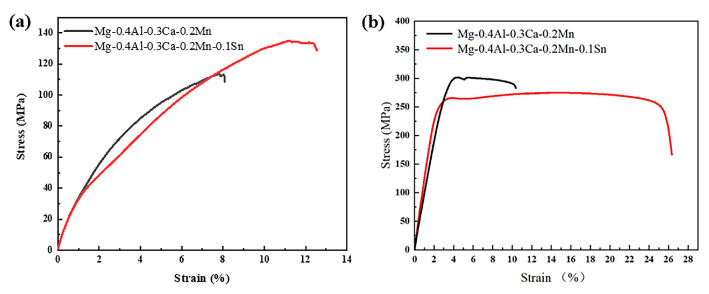
Stress–strain curve of homogenized and extruded Mg-0.4Al-0.3Ca-0.2Mn-xSn alloys (x = 0, 0.1 wt.%): (**a**) homogenized; (**b**) extruded.

The tensile properties of the homogenized and extruded Mg-0.4Al-0.3Ca-0.2Mn-xSn alloys are presented in Figure 11 and summarized in Table 3. In the homogenized state (Figure 11a), the addition of 0.1 wt.% Sn improves both the UTS (from 107 MPa to 132 MPa) and elongation (from 8.1% to 12.5%), which can be attributed to the solid solution strengthening and the modification of as-cast secondary phases.

**Figure 12:**
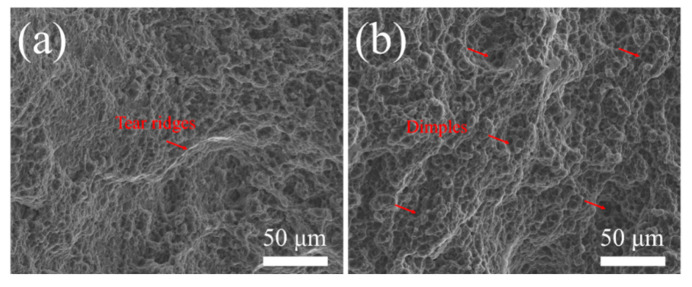
Fracture morphologies of extruded Mg-0.4Al-0.3Ca-0.2Mn-xSn alloys (x = 0, 0.1 wt.%): (**a**) x = 0; (**b**) x = 0.1.

For the extruded alloys (Figure 11b), a remarkable strength–ductility synergy is achieved through trace Sn microalloying. The Sn-free alloy (x = 0) exhibits a higher YS of 283 ± MPa but a limited elongation of 12.9 ± 5.4%. In contrast, the 0.1Sn alloy (x = 0.1) shows a slightly lower YS (255 ± 7 MPa) but a doubled elongation of 26.3 ± 1.3%. The high YS in the x = 0 alloy is primarily derived from the strong basal fiber texture (intensity of 20.86, as shown in Figure 12a), which acts as a “hard orientation” that significantly resists plastic flow along the ED.

In addition, in Mg-0.4Al-0.3Ca-0.2Mn alloy, the dense fine particles with small spacing crushed have an inhibitory effect on the progress of DRX. Figure 6 shows the tensile fracture morphology of the extruded Mg-Al-Ca-Mn-xSn (x = 0, 0.1 wt.%) alloy. Compared with Figure 12b, the dimples in Figure 12a alloy are relatively shallow and have obvious tearing edges, indicating that after adding Sn, the plasticity is better.

The dramatic enhancement in ductility for the 0.1Sn alloy is intrinsically linked to the texture weakening and the formation of a heterogeneous bimodal structure. The reduction in texture intensity (from 20.86 to 9.99) and the corresponding increase in SF (Figure 9) effectively lower the CRSS for basal and non-basal slips. This “texture softening” effect facilitates more homogeneous plastic deformation and reduces stress concentration at grain boundaries. When the alloy is subjected to tensile or compressive load, the soft DRXed grains begin to deform plastically first. However, their deformation is constrained by the hard, un-DRXed skeleton. This is primarily manifested in the following three aspects:

(1) Dislocation Pile-up: Dislocations in the soft grains glide until they encounter the phase boundaries or grain boundaries of the hard domains. They cannot easily cross into the hard domain due to the high dislocation density and orientation mismatch.
(2) Back Stress Generation: These pile-ups generate a long-range elastic back stress (σ_b_) in the soft grains, which opposes the motion of further dislocations. This effectively “hardens” the soft grains rapidly [13].
(3) Forward Stress: Simultaneously, a forward stress (σ_f_) is concentrated onto the hard grains. This stress concentration eventually becomes high enough to activate slip systems in the hard grains, forcing them to deform plastically.

This partitioning of stress and strain between the soft and hard domains leads to an extraordinarily high strain hardening rate. The material can sustain increasing loads (high strength) while the soft domains continue to deform (high ductility), delayed by the stabilizing effect of the back stress [32]. Therefore, trace Sn microalloying plays a dual role: it refines the microstructure by stimulating DRX and “softens” the hard-oriented grains by weakening the basal texture. This dual effect results in a more homogeneous distribution of internal strain and a lower threshold for activating multiple slip systems, thereby providing the possibility of achieving excellent plasticity while maintaining high strength.

## 4. Conclusions

In this study, a high-performance Mg-0.4Al-0.3Ca-0.2Mn-0.1Sn alloy with a tailored heterogeneous bimodal structure was successfully fabricated. The critical role of trace Sn microalloying on microstructure evolution, crystallographic orientation, and mechanical properties was systematically investigated. The main conclusions are as follows:

(1) Trace Sn addition (0.1 wt.%) effectively promotes the DRX process during hot extrusion. The formation of uniformly distributed CaMgSn phases serves as a potent site for particle-stimulated nucleation, resulting in a significantly refined DRXed grain size (from 2.66 μm to 2.11 μm) and an increased DRXed area fraction.
(2) Both alloys exhibit a bimodal grain structure consisting of fine DRXed grains and coarse un-DRXed bands. The 0.1Sn alloy demonstrates a more optimized heterogeneous configuration, where the high-dislocation-density un-DRXed regions (confirmed by KAM analysis) act as a high-strength skeleton, while the refined DRXed regions facilitate strain relaxation and toughening.
(3) Trace Sn microalloying induces a dramatic texture-weakening effect, reducing the maximum texture intensity from 20.89 to 9.99. This “texture softening” leads to a substantial increase in SF for basal, non-basal <a>, and <c+a> pyramidal slip systems. The enhanced activity of multiple slip modes, especially <c+a> slips, effectively accommodates c-axis strain and promotes homogeneous deformation.
(4) A remarkable balance between strength and ductility was achieved in the extruded 0.1Sn alloy. Compared to the Sn-free alloy, the 0.1Sn alloy exhibits a doubled elongation (from 12.9% to 26.3%) while maintaining a high UTS (274 MPa). This synergy is primarily governed by the combination of grain boundary strengthening and the enhanced plastic stability provided by the weakened basal texture and heterogeneous structure.

## Figures and Tables

**Table 1:** EDS results of the second phase particles marked by arrows in Figure 2.

Label	Mg (At.%)	Al (At.%)	Ca (At.%)	Mn (At.%)	Sn (At.%)	Phase
A	2.0	45.5	0.1	52.4	0	Al_8_Mn_5_
B	74.0	11.6	14.3	0	0	(Mg, Al)_2_Ca
C	78.3	9.0	12.6	0.1	0	(Mg, Al)_2_Ca
D	73.8	0.3	11.8	0	14.1	CaMgSn
E	69.7	0.4	13.5	0	16.3	CaMgSn
F	67.3	0.4	13.6	0.2	18.4	CaMgSn
G	74.7	0.4	11.3	0	13.6	CaMgSn
H	76.3	7.4	15.8	0	0.5	(Mg, Al)_2_Ca

**Table 2:** EDS results of extruded Mg-0.4Al-0.3Ca-0.2Mn-xSn alloys (x = 0, 0.1 wt.%).

Label	Mg (At.%)	Al (At.%)	Ca (At.%)	Mn (At.%)	Sn (At.%)	Phase
A	78.4	13.9	7.7	0	0	(Mg, Al)_2_Ca
B	84.7	6.4	8.8	0	0	(Mg, Al)_2_Ca
C	46.5	26.3	6.3	20.9	0	Al_8_Mn_5_
D	71.7	5.0	13.8	0	9.5	CaMgSn
E	76.9	2.1	10.9	0.1	10.0	CaMgSn
F	64.9	6.8	18.1	0	10.2	CaMgSn
G	75.7	10	13.5	0.1	0.7	(Mg, Al)_2_Ca

**Table 3:** Tensile properties of homogenized and extruded alloys.

Alloy	YS (MPa)	UTS (MPa)	EL (%)
Homogenized (x = 0)	-	107 ± 5	8.1 ± 1.7
Homogenized (x = 0.1)	-	132 ± 2	12.5 ± 0.6
Extruded (x = 0)	283 ± 6	303 ± 2	12.9 ± 5.4
Extruded (x = 0.1)	255 ± 7	274 ± 1	26.3 ± 1.3

## Data Availability

The original contributions presented in this study are included in the article. Further inquiries can be directed to the corresponding author.
